# Evaluation of in vitro toxicity of peptide (*N*-acetyl-Leu-Gly-Leu-COOH)-substituted-β-cyclodextrin derivative, a novel drug carrier, in PC-12 cells

**DOI:** 10.1186/2008-2231-21-75

**Published:** 2013-12-20

**Authors:** Hamid Reza Sadeghnia, Faezeh Vahdati Hassani, Hamid Sadeghian, Maryam Miandehi, Farzin Hadizadeh, Gholamreza Karimi

**Affiliations:** 1 Neurocognitive Research Center and Department of Pharmacology, School of Medicine, Mashhad University of Medical Sciences, Mashhad, Iran; 2 Medical Toxicology Research Center, Pharmacy School, Mashhad University of Medical Sciences, Mashhad, Iran; 3 Antimicrobial Resistance Research Center, Department of Laboratory Sciences, Mashhad University of Medical Sciences, Mashhad 91967-73117, Iran; 4 Pharmacy School, Mashhad University of Medical Sciences, Mashhad, Iran; 5 Biotechnology Research Center, Pharmacy School, Mashhad University of Medical Sciences, Mashhad, Iran

**Keywords:** Hepta-(*N*-acetyl-Leu-Gly-Leu)-β-CD, Lipid peroxidation, DNA damage, Oxidative stress, PC-12 cells

## Abstract

**Background:**

Cyclodextrins (CDs) have been shown to improve physicochemical and biopharmaceutical properties of drugs when low solubility and low safety limit their use in the pharmaceutical field. Recently, a new amphiphilic peptide-substituted-β-CD, hepta-(*N*-acetyl-Leu-Gly-Leu)-β-CD (hepta-(*N*-acetyl-LGL)-β-CD), is developed which exhibited good solubility, strong inclusion ability and an appropriate average molecular weight. However, there is limited information available about its toxic effects. This study was designed to evaluate cytotoxic effects of the hepta-(N-acetyl-LGL)-β-CD (50, 200, 400, and 800 μg/ml) on rat pheochromocytoma PC-12 cells.

**Results:**

A significant reduction of cell viability with IC_50_ values of 1115.0 μg/ml, 762.4 μg/ml, and 464.9 μg/ml at 6, 12, and 24 h post-treatment, respectively, as well as increased lipid peroxide levels and DNA damage were observed.

**Conclusions:**

In conclusion, hepta-(*N*-acetyl-Leu-Gly-Leu)-β-CD exhibit significant toxic properties at high concentrations, probably through induction of oxidative stress and genotoxicity.

## Background

Toxic potential, lack of efficacy, and especially low aqueous solubility, that lead to insufficient therapeutic concentration in physiological fluids, are main causes of drug delivery failure for approximately 40% of current and 90% of new drugs in the market according to biopharmaceutical classification system [1]. In order to enhance aqueous solubility and membrane penetration of drugs, numerous methods including prodrugs, physical methods, and water-soluble nanocarrier systems such as liposomes, microemulsions, and polymeric nanoparticles have been provided [1,2]. One of the beneficial techniques to improve the water solubility, bioavailability, and stability of drug formulations is the use of cyclodextrins (CDs) and chemically modified ones [3-6]. CDs are hydrophilic or relatively lipophilic oligomers that are produced by enzymatic degradation of starch. There are three major CDs: α-CD, β-CD, and γ-CD, which are composed of 6, 7 or 8 α -(1,4)-linked glucopyranose glucose residues, respectively, arranged in a cone-shaped, formed a hydrophilic outer surface and a somewhat lipophilic cavity [2,7]. Because of their use as an excipient in pharmaceuticals, many studies have evaluated the safety of natural CDs and their derivatives in several in vitro and animal models [8,9]. Genotoxicity test of CDs indicated that none of them are genotoxic and mutagenic. However, exocrine acinar cell neoplasia was observed in some studies [9]. The hemolytic effect, one of the main disadvantages of CDs, also was evaluated in different studies and correlated with their effect to solubilize membrane cholesterol [10-12]. Recently, new amphiphilic β-CDs were designed by substitution of peptide chains on to the primary hydroxyl groups through ester bond formation between the carboxyl group of *N*-acetylated residues and C-6 of β-CD [13]. In the present study, we investigated the toxicity of hepta-(*N*-acetyl-Leu-Gly-Leu)-β-CD (one of the peptide-substituted-β-CD) designed by Seyedi et al. [13] in PC-12 cells. Furthermore, lipid peroxidation and possible DNA damage were also evaluated.

## Methods

### Chemicals

Hepta-(*N*-acetyl-LGL)-β-CD provided by Seyedi et al. [13]; PC-12 cells purchased from Pasteur Institute, Tehran, Iran; Dulbecco’s Modified Eagle’s Medium (DMEM) 4.5 mg/ml glucose, 3-(4,5-dimethylthiazol-2-yl)-2,5-diphenylterazolium bromide (MTT), penicillin, and streptomycin from Gibco, USA; L-glutamine, fetal bovine serum (FBS), trypsin, and dimethyl sulfoxide (DMSO) from Merck, Germany; low and normal-melting temperature agarose (LMA and NMA, respectively) from Biogen, USA; ethidium bromide (EB), thiobarbituric acid (TBA), hydrochloric acid (HCl), trichloroacetic acid (TCA), Bicinchoninic Acid (BCA) Kit, sodium chloride (NaCl), ethylenediaminetetracetic acid disodium salt (Na_2_EDTA), tris-(hydroxymethyl)-aminomethane (Tris–HCl), sodium N-lauroyl-sarcosinate, Triton X-100, and sodium hydroxide (NaOH) from Sigma-Aldrich, Germany.

### Cell culture

PC-12 cells were cultured with DMEM (high glucose, 4.5 mg/ml glucose) which was supplemented with 10% FBS, 2 mM of L-glutamine, 100 U/ml of penicillin and 100 μg/ml of streptomycin and maintained in a humanized atmosphere (90%) containing 5% CO_2_ at 37°C. ATCC instructions were used to perform subculture. Throughout the experiment, after 2–3 days at 80-90% confluency, cells were plated on sterile poly-L-lysine coated 96-well microplates (5000 cells/well) and were used 24 h later.

### Cell survival assay

After 24 h seeding, cells were treated with different concentrations (50, 200, 400, and 800 μg/ml) of hepta-(*N*-acetyl-LGL)-β-CD and incubated for 6, 12, and 24 h. MTT assay was used to determine cell viability [14]. Briefly, MTT (5 mg/ml) was added to each well and cells were cultured for 3 h at 37°C to allow the reaction to proceed. Then, the media was removed and the reduced formazan crystals were dissolved in 100 μl DMSO. The absorbance of each well was read at 550 nm using a microplate reader (VICTOR™ X3 Multilabel Plate Reader, Perkin Elmer, Finland). For each concentration three wells were prepared and each plate was run in triplicate.

### Alkaline single cell gel electrophoresis or comet assay

Comet assay was performed as a three-layer procedure under alkaline (pH > 13) conditions [15] with some modifications. For a typical experiment, cells were seeded in sterile poly-L-lysine coated 12-well plates (10^6^ cells/well) and incubated at 37°C in 90% humanized atmosphere, 5% CO_2_ for 24 h. The cultured cells were then washed with PBS and exposed to different concentrations of hepta-(*N*-acetyl-LGL)-β-CD (50, 200 and 800 μg/ml) for 6, 12, and 24 h. After that, cells were trypsinized, centrifuged at 3000 g for 4 min. 50 μl of cell pellet was suspended in 300 μl LMP agarose 1%, which was dissolved in PBS, and kept at 37°C in water bath. 150 μl of cell/agarose mixture was spread on a conventional 26 mm × 76 mm microscope slides precoated with 100 μl of NMP agarose 1% and covered with a top layer of NMP agarose 1%. Before the LMP agarose solidified, a cover slip was added. Subsequently, the embedded cells were placed in a lysis solution (2.5 M NaCl, 100 mM Na2EDTA, 10 mM Tris–HCl, 1% sodium N-lauroyl-sarcosinate, 1% Triton X-100, and 10% DMSO; pH 10) for 24 h at 4°C. The following day, the slides were placed in a horizontal electrophoresis tank, immersed and left in fresh cold alkaline electrophoresis buffer solution (300 mM NaOH, 1 mM Na_2_EDTA; pH > 13) for 40 min at 4°C. Electrophoresis was performed using the same solution for 40 min by applying an electric field of 24 V and adjusting the current to 300 mA. Finally, the slides were washed three times with neutralization buffer (400 mM Tris–HCl buffer; pH 7.5). After washing with deionized water, the slides were placed at room temperature for 48 h to dry and then stained with 50 μl of EB (20 μg/ml). Three wells were treated for each experimental group and each experiment was repeated three times.

### Evaluation of DNA damage

Fluorescence microscope (Eclipse TE2000, Nikon, Japan) applying 520–550 nm excitation filter and 580 nm barrier filter was used to visualize EB stained slides (magnification 400×). Comet assay software project (CASP) was applied to determine percentage of tail DNA of each nucleoid. One hundred nucleoids for each concentration (50 per slide) were analyzed for quantification of DNA damage.

### Measurement of malondialdehyde (MDA)

PC-12 cells were cultured in sterile poly-L-lysine coated 12- well plates (10^6^ cells/well) according to the procedures described above and exposed to the sample solutions (50, 200, and 800 μg/ml). The malondialdehyde (MDA) content, as a measure of lipid peroxidation, was assayed using the protocol described by Mihara et al. with some modifications [16]. After treatment for 6, 12, and 24 h, the cells were homogenized. Next, 2 mL of 0.7% TBA, 0.25 M HCl, and 15% TCA mixture was added to the homogenate, vortexed well and incubated in boiling water for 20 min following by centrifugation at 3000 g for 5 min at 4°C. The absorbance of the resulting supernatants was then immediately measured for the levels of MDA at 530 and 550 excitation and emission wavelength, respectively. Finally, the total protein content of the samples was determined by BCA Kit to normalize the levels of MDA. MDA levels were expressed in nmol/mg protein.

### Statistical analysis

All data are expressed as mean ± SEM. One Way Analysis of Variance (ANOVA) followed by Tukey or Bonferroni’s post-hoc test using GraphPad InStat version 3.00 (GraphPad Software, San Diego, California, USA) was used to perform statistical analysis. *P* value less than 0.05 was considered to be statistically significant.

## Results

### Hepta-(*N*-acetyl-LGL)-β-CD-induced cytotoxicity

**Figure 1:**
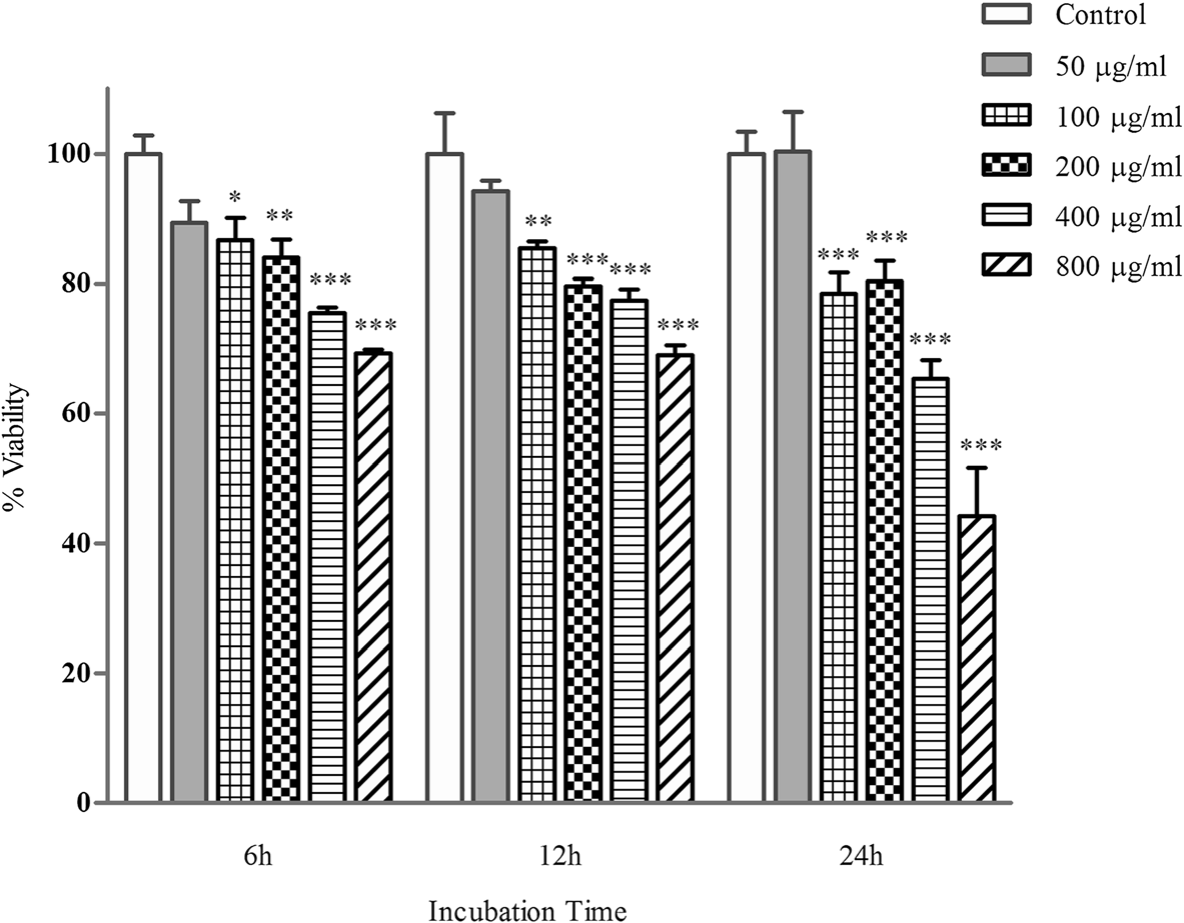
**Effects of different concentrations of hepta-(*N*-acetyl-LGL)-β-CD on PC-12 cells viability.** Cells were incubated with different concentrations of the CD for 6, 12, and 24 h. Cell viability was determined by MTT assay. Data were shown as mean percentage of the treated cells ± SEM for 3 wells per group (repeated 3 times) vs. untreated cells (control) as 100% (*P < 0.05, **P < 0.01, ***P < 0.001).

The effects on cell viability of PC-12 cells were exposed to different concentrations of hepta-(*N*-acetyl-LGL)-β-CD examined using MTT assay. Figure 1 shows that 6, 12, and 24 h cell incubation with hepta-(*N*-acetyl-LGL)-β-CD induced a significant reduction of PC-12 cell viability at 100 μg/ml and higher concentrations vs. control (untreated cells) (*p* < 0.05). However, the effect was not time-dependent. The highest percentage of cell death was recorded when PC-12 cells incubated with the highest concentration of the CD (800 μg/ml) for 24 h (44.24 ± 7.41% vs. control 100%).

### Evaluation of DNA damage by comet assay

**Figure 2:**
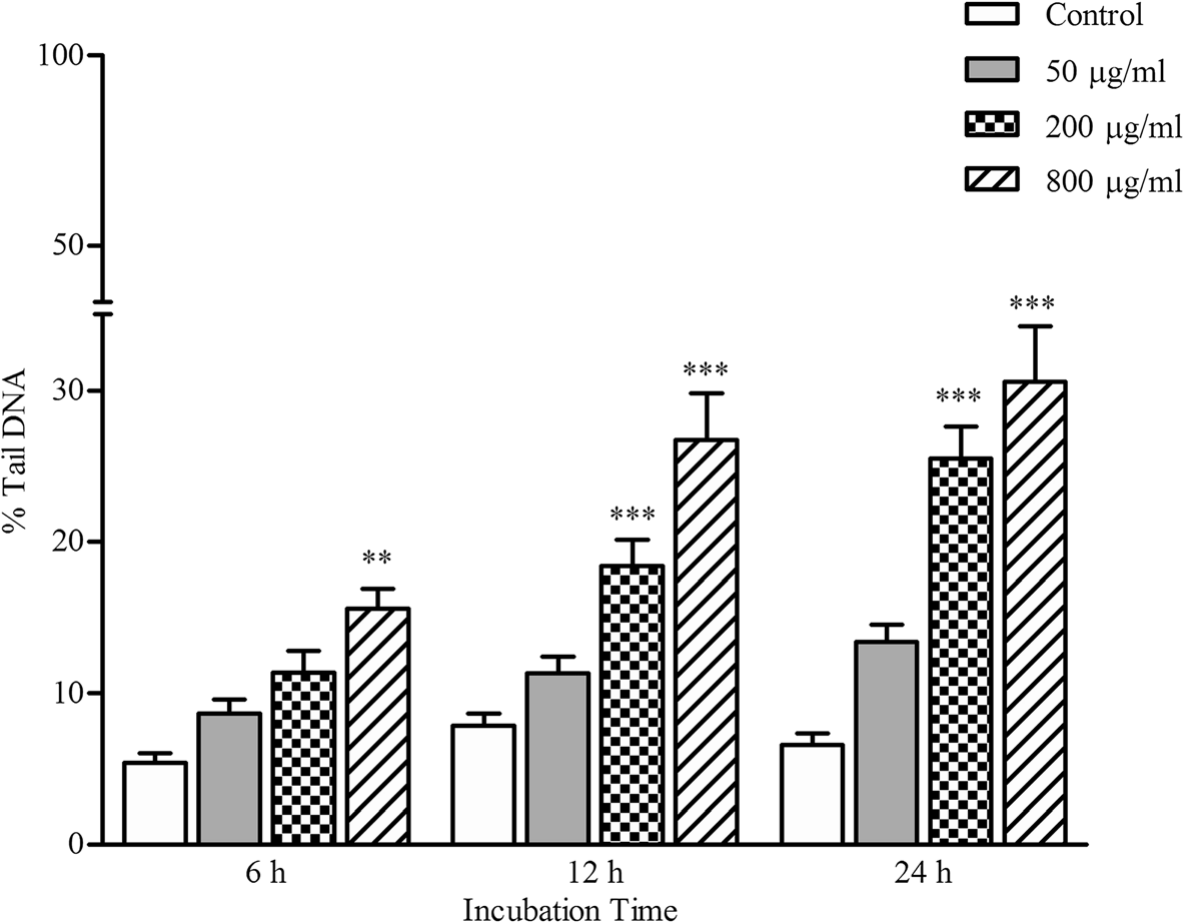
**DNA damage in PC-12 cells were exposed to different concentrations of hepta-(*N*-acetyl-LGL)-β-CD for 6, 12, and 24 h.** Data were shown as mean percentage of tail DNA of treated cells ± SEM for 3 wells per group (repeated 3 times) vs. untreated cells (control) as 100% (**P < 0.01,***P < 0.001).

**Figure 3:**
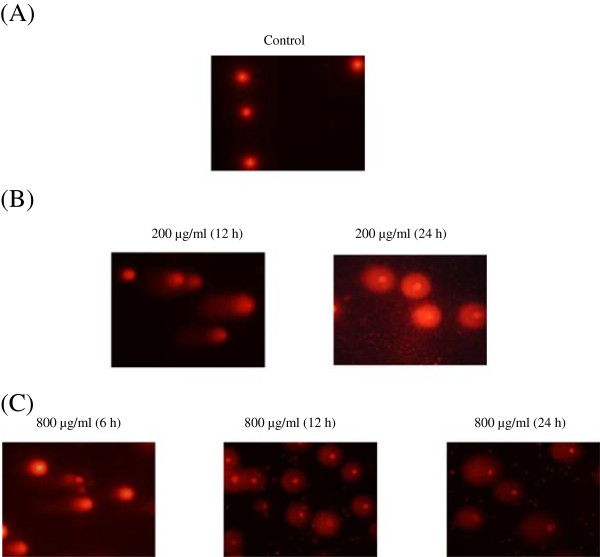
**Representative photomicrographs of a comet assay showing genotoxic effects of hepta-(*N*-acetyl-LGL)-β-CD. A**: the untreated PC-12 cells (control); **B** and **C** showing DNA damage in PC-12 cells treated with 200 and 800 μg/ml concentrations respectively after 6,12, and 24 h exposure.

As shown in Figure 2, PC-12 cells incubated with different concentrations of hepta-(*N*-acetyl-LGL)-β-CD exhibited significantly higher DNA damage (*p* < 0.05) than the control (untreated cells) but this effect was not time-dependent. The highest DNA damage was observed at 800 μg/ml concentration for all incubation times (6 h: 15.59 ± 1.29%; 12 h: 26.74 ± 3.06%; 24 h: 30.58 ± 3.65%) followed by 200 μg/ml for 12 h (18.37 ± 1.75%) and 24 h (25.47 ± 2.14%) incubation (*p* < 0.05). Significant induction of DNA damage in PC-12 cells by hepta-(*N*-acetyl-LGL)-β-CD concentrations was presented in Figure 3.

### Effects of hepta-(*N*-acetyl-LGL)-β-CD on MDA

**Figure 4:**
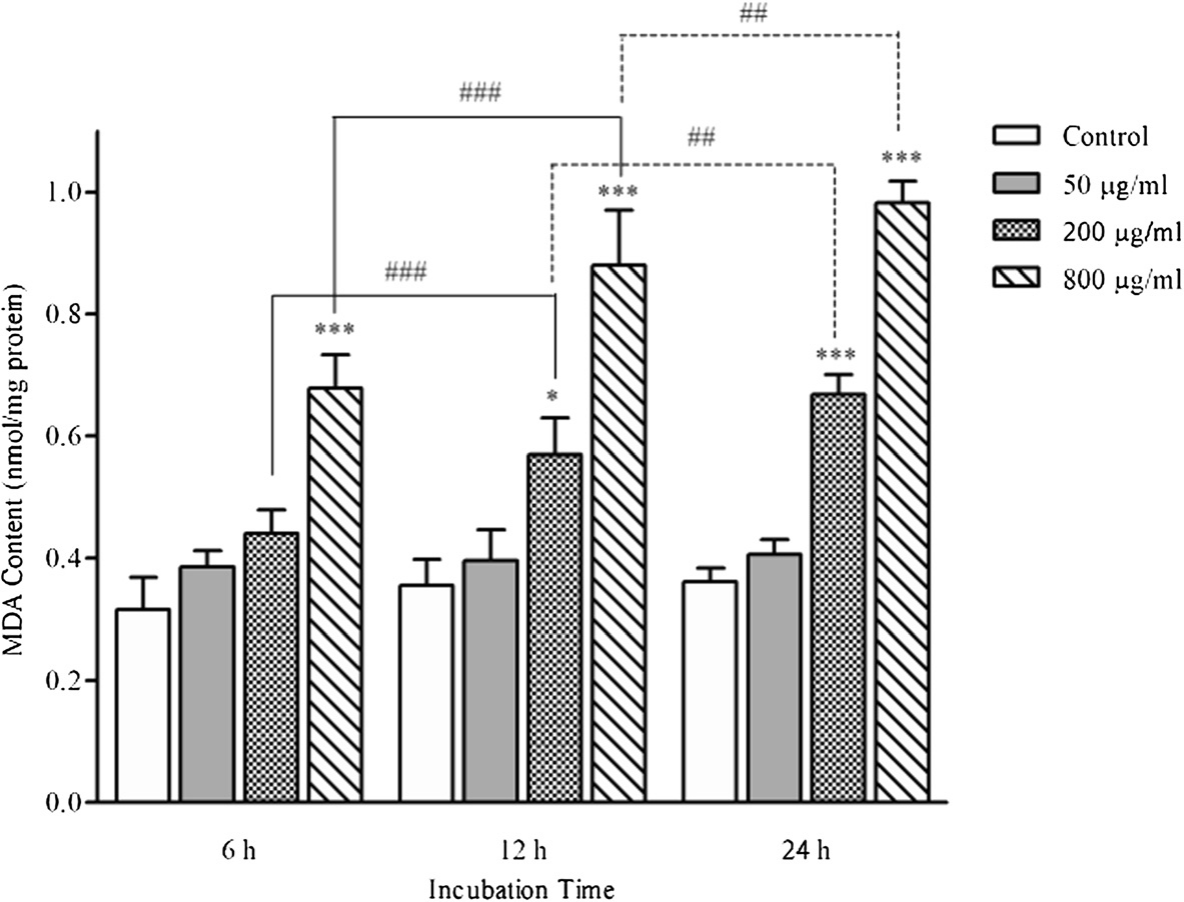
**MDA contents in PC-12 cells After treatment with different concentrations of hepta-(*N*-acetyl-LGL)-β-CD for 6, 12, and 24 h.** Data were shown as mean MDA contents of treated cells ± SEM for 3 wells per group (repeated 3 times) vs. untreated cells (control) (*P < 0.05, ***P < 0.001). ##*p* < 0.01, ###*p* < 0.001, comparison among different incubation times.

As shown in Figure 4, after PC-12 cells were exposed to different concentrations of hepta-(*N*-acetyl-LGL)-β-CD, changes in contents of MDA were observed. Treatment with the CD Significantly increased MDA levels after 6 h (0.68 ± 0.054 nmol/mg protein), 12 h (0.88 ± 0.090 nmol/mg protein), and 24 h (0.98 ± 0.036 nmol/mg protein) incubation with 800 μg/ml dose and 12 h (0.57 ± 0.060 nmol/mg protein) and 24 h (0.67 ± 0.032 nmol/mg protein) incubation with 200 μg/ml dose. When the CD concentration was increased from 200 μg/ml to 800 μg/ml, MDA levels increased in a time-dependent manner.

## Discussion

**Figure 5:**
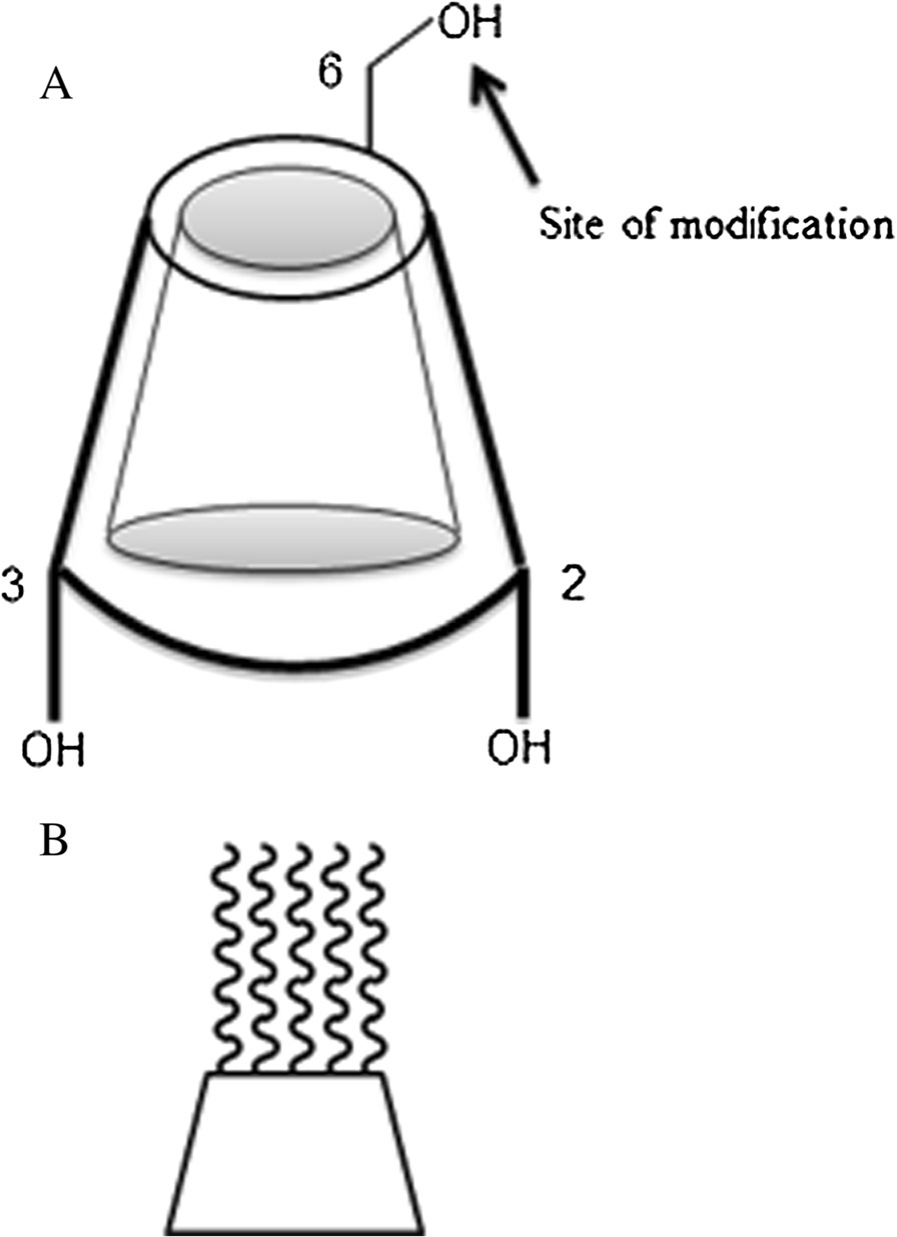
**Schematic presentations of natural CD structure and modification sites.** hepta-(*N*-acetyl-LGL)-β-CD synthesized by grafting peptide chains on to the primary hydroxyl group via esterification **(A)**. Schematic presentation of the amphiphilic cyclodextrin used in our work **(B)**.

**Figure 6:**
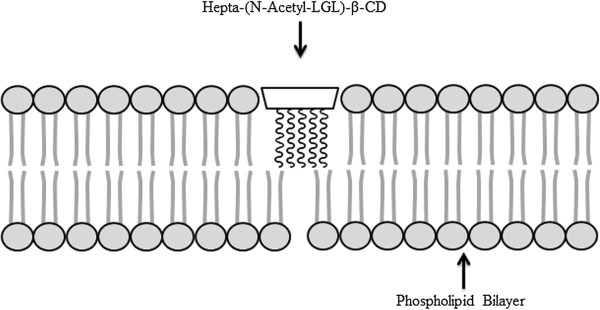
**Schematic presentation of, hepta-(*N*-acetyl-LGL)-β-CD interaction with cell membranes.**

In the present study, we evaluated the cytotoxic effects of hepta-(*N*-acetyl-LGL)-β-CD on PC-12 cells. The most toxic effects observed at 800 μg/ml concentration for 24 h incubation. Our results showed that the CD effects on cell viability may be the consequence of interaction between the CD and cellular lipids (MDA content) and DNA content (comet assay), possibly through strongly lipid peroxidation and DNA damage. Comet assay is a fast, simple, sensitive and cheap technique to investigate DNA damage in all mammalian cell types [17]. Although genotoxicity in other in vivo and in vitro evaluations of α, β and γ-cyclodextrin and their alternatives was negligible [8,9], we showed that incubation with high doses of hepta-(*N*-acetyl-LGL)-β-CD induced obvious DNA damage. Malondialdehyde (MDA) is a consequence of decomposition of certain primary and secondary lipid peroxidation products [18]. Significant increase in the levels of MDA indicated that hepta-(*N*-acetyl-LGL)-β-CD induced oxidative damage in PC-12 cells and this effect was time-dependent in higher doses. The ability of CDs to destruct basic membrane and internal cell components like solubilizing cholesterol were reported and correlated with their cytotoxic effects (e.g. hemolytic activity) [10-12]. These experimental observations may be the results of CD’s structure. It was demonstrated that the cytotoxic effects of different β-CD derivatives depend on their ability to extract cholesterol from the cell membranes. This activity was strongly affected by inserting diverse substitutions on CD’s structure [19]. In hepta-(*N*-acetyl-LGL)-β-CD, an amphiphilic peptide-β-cyclodextrin, the peptide chains would make an intimate contact with the lipid bilayer because of its phospholipid like shape. So it can disturb membrane integrity and its toxicity probably is a result of this interaction (Figures 5 and 6). Also it was mentioned that nanoparticles due to their miniscule size can more easily penetrate various biological barriers such as cell membranes when compared to non-nanoparticles and this lead to toxicity; however, there is not enough convincing reasons [20].

## Conclusions

In conclusion, we revealed that new β-CD derivative, hepta-(*N*-acetyl-LGL)-β-CD, is a potential cytotoxic agent. The results showed that measurement of lipid peroxidation and DNA damage contents may be simple markers to evaluate the cytotoxicity of novel β-CD derivatives and provide information for future studies to find a safer peptide substitution or to alter the peptide structure. However, potential safety hazards of this carrier in pharmaceutical formulations need further in vivo studies.

## Competing interest

The authors declare that they have no competing interests.

## Authors’ contributions

GK designed the study. HRS and HS were the supervisors. HS carried out the synthesis of hepta-(*N*-acetyl-LGL)-β-CD. MM and FVH participated in doing the experiments and helped to draft the manuscript. FH had role in writing the discussion. All authors read and approved the final manuscript.
